# *Huperzia serrata* Extract ‘NSP01’ With Neuroprotective Effects-Potential Synergies of Huperzine A and Polyphenols

**DOI:** 10.3389/fphar.2021.681532

**Published:** 2021-08-30

**Authors:** N. Callizot, ML Campanari, L Rouvière, G Jacquemot, A. Henriques, E Garayev, P. Poindron

**Affiliations:** ^1^ Neuro-Sys SAS, Neuro-Pharmacology Department, Gardanne, France; ^2^ Neuralia SAS, Gardanne, France

**Keywords:** huperzia serrata, neuroprotective activity, NSP01, huperzine A, caffeic acid, ferulic acid, synergistic combination, ERK pathway

## Abstract

*Huperzia serrata* (Thunb.) Trevis is widely used in traditional asiatic medicine to treat many central disorders including, schizophrenia, cognitive dysfunction, and dementia. The major alkaloid, Huperzine A (HA), of *H. serrata* is a well-known competitive reversible inhibitor of acetylcholinesterase (AChE) with neuroprotective effects. Inspired by the tradition, we developed a green one-step method using microwave assisted extraction to generate an extract of *H. serrata*, called NSP01. This green extract conserves original neuropharmacological activity and chemical profile of traditional extract. The neuroprotective activity of NSP01 is based on a precise combination of three major constituents: HA and two phenolic acids, caffeic acid (CA) and ferulic acid (FA). We show that CA and FA potentiate HA-mediated neuroprotective activity. Importantly, the combination of HA with CA and FA does not potentiate the AChE inhibitory property of HA which is responsible for its adverse side effects. Collectively, these experimental findings demonstrated that NSP01, is a very promising plant extract for the prevention of Alzheimer’s disease and memory deficits.

## Introduction

Alzheimer’s disease (AD) is the commonest cause of dementia in the world with huge implications for individuals and society. Although the etiology of AD remains uncertain, the chronic memory loss and cognitive decline are thought to be due, at least in part, to a progressive deposition of senile plaques and neurofibrillary tangles in cerebral cortex and basal forebrain (the amyloid and tangle cascade hypothesis) (Jan et al., 2010; Callizot et al., 2013). Other relevant factors, including cholinergic dysfunction, neuroinflammation, oxidative stress, mitochondria dysfunctions, disturbance of neuronal plasticity, age-related loss of sex hormones are important and contribute to the understanding of AD pathology (Richter et al., 2014; Heneka et al., 2015; Jang and Chung, 2016; Swerdlow, 2018).

Despite the growing population of patients affected of AD, only four drugs are currently approved to treat the cognitive symptoms of AD in European Union; these include three cholinesterase inhibitors (donepezil, galantamine, and rivastigmine) and one *N*-methyl-d-aspartate (NMDA) receptor antagonist (Hyde et al., 2013; Tan, 2014). However, none of them profoundly affects the advancement of the disease.

Some plants belonging to the Lycopodiaceae family, such as *H. serrata* (Thunb.) Trevis, *H. squarrosa* (Forst.) Trevis, *Lycopodium complanatum* L. and *L. cernua* L., are used in traditional Chinese and Vietnamian medicine for thousands of years to treat contusions, strains, schizophrenia and memory dysfunction (Ma et al., 2006; Ma et al., 2007; Chuong et al., 2014; ; Petelor, 1952). Their use by aqueous decoction is considered as “traditional extract” (Obringer, 2006). *H. serrata* (shé zú shí shān in chinese) is a perennial herbaceous plant (15–40 cm) close to ferns, growing in wetlands and forests in most of China and in the northern part of Vietnam, a region which was a province of the Chinese Empire, under the name of Jiaozhi (from 111 BC up to 939 AD) (Maspero, 1967) so as the present distinction between China and Vietnam is more political than botanical. *H. serrata* is widely distributed in southern Asia, in India and north America (Ferreira et al., 2016). It is used as aqueous infusion for its memory-improving properties (Howes et al., 2003; Howes and Houghton, 2003; Cuthbertson et al., 2012; Vallejo et al., 2013). It was reported to enhance cognition and was recommended for the treatment of senile dementia. HA, the major alkaloid of *H. serrata*, has been proved to be a powerful, highly specific, and reversible acetylcholinesterase (AChE) inhibitor with a long duration of action and the ability to cross the blood–brain barrier (Jia Sen et al., 1986; Tang et al., 1986; Tang_De_Sarno et al., 1989; Ohba et al., 2015). Its neuroprotective actions include the regulation of β-amyloid precursor protein metabolism, the protection against Aβ-mediated oxidative stress, apoptosis and mitochondrial dysfunction, as well as anti-inflammatory effects (Zhang and Tang, 2006; Zhang et al., 2008; Qian and Ke, 2014). Also, clinical reports from China, where an estimated 100,000 people have been treated with HA, have showed HA low toxicity (Skolnick, 1997; Chiu and Zhang, 2000). Finally, HA was shown to be an effective and safe drug in alleviating cognitive dysfunctions in AD patients and aged subjects with benign senescent forgetfulness (Xu et al., 1997; Wang et al., 2006a; Zhang et al., 2008; Xu et al., 2012; Yang et al., 2013; Xing et al., 2014).

Inspired by the tradition, we applied a green single fast track step process using microwave assisted extraction in order to generate an extract of *H. serrata*, called NSP01 (acronymous term from NeuroSys Plants N°1).

We characterized the chemical profile of the green extract and identified three major components synergistically acting and supporting the neuropharmacological properties: HA, CA and FA. We tested the effect of NSP01 and HA/CA/FA mixture and shown that are protective in primary neuronal cultures injured with glutamate or Aβ oligomers. In particular we shown that HA, CA and FA act in synergy without potentiate AChE inhibition. Finally, we propose a NSP01 mechanism of action involving ERK pathway.

## Materials and Methods

**Plant material:** The plant name has been checked with http://www.theplantlist.org on April 16, 2019 (“Huperzia serrata (Thunb.) Trevis. — The Plant List,” 2012). The plant has been purchased by “Anhui Highkey import and export co” and the product was accompanied by its phytosanitary certificate. The plant material (aerial part) of *H. serrata* was collected in Bi Doup, Vietnam in April 2013, then sorted, washed with water and brushed to remove impurities. To remove microbial contaminations, the plant was treated with a chloramine B solution (5%) during 15 min. Then, the plant was chopped into small pieces and dried for 12 h, at 40–45°C, until desiccated. The grinded herbal drug was conditioned in hermetic plastic bag and protected from light. A voucher (NSP01-01) of the harvested specimen was kept, identified and confirmed by Prof. Tran Hop (Department of Botany, Ho Chi Minh City, Vietnam).

**Plant extracts:** The traditional extract (NSP01-TE) was prepared by extraction of air-dried plant material with demineralized water by decoction for 30 min in a boiling water bath (1:15, vegetal mass:solvent volume). After cooling down to room temperature, the crude extract was filtered through cotton sheet. The filtered solution was frozen at −20°C and freeze-dried to produce dried powder.

The microwave-assisted extract (NSP01) was prepared under atmospheric pressure by extraction of air-dried plant material with demineralized water for 30 min at 200 W using microwave assisted extractor Milestone Ethos EX (Milestone, Sorisole, Italy) (1:15, vegetal mass:solvent volume). After extraction, the mixture was centrifuged at 11,952 x g for 5 min at 25°C then filtrated through cotton sheet, frozen at −20°C and freeze-dried to produce dried powder.

### Analytical Method

**Sample preparation:** 100 µL of DMSO were added to 10 mg of dried extract in a 5 ml volumetric flask, and the mixture was sonicated for 2 min. Two hundred µL of water were then added and the solution was sonicated for 2 min. Volume was adjusted to 5 ml with water and the mixture was sonicated for 2 min. Final solution was then filtered through a 0.2 µm filter (⌀ 13 mm, GHP) into an HPLC vial.

### UHPLC-MS Method

• *UHPLC-MS method for identification*

UHPLC-DAD-ESI-MS/MS (Ultra High-Performance Liquid Chromatography–Diode array detection–Electrospray Ionization Mass Spectrometry) analysis was performed on Thermo Ultimate 3,000 Ultra High-Performance Liquid chromatograph with quaternary pump system, connected to a DAD detector (200–400 nm) piloted by Hystar software and to a Bruker (Palaiseau, France) UHR-qQTOF (Ultra-High Resolution Qd-Time-Of-Flight) system with an ESI interface. Chromatographic separation was achieved using Thermo Acclaim RSLC 120 C18 column (150 × 2.1 mm, 2.2 µm; Thermo Fisher Scientific, Waltham, MA, United States), operated at 45°C. Water acidified with 0.1% of formic acid (v/v) as solvent A (pH 2.7) and methanol acidified with 0.1% of formic acid (v/v) as solvent B were used for the gradient elution at 0.4 ml/min. The gradient program was: 5–35% B (from 0 to 14.5 min), 35–95% B (from 14.5 to 14.6 min), 95% B (from 14.6 to 15.6 min), 95 to 5% B (from 15.6 to 15.7 min), 5% B (from 15.7 to 17.0 min), injection volume of 20 µL and UV detection wavelength set at 310 nm. For compounds identification, mass spectrometry parameters were as follows: positive and negative ionization modes, spray voltage, 3.5 kV; sheath gas flow rate, 5.8 psi; auxiliary gas flow rate, 4 L/min, capillary temperature, 200°C and spectra acquisition rate, 1 Hz. Fragmentation energy for MS^2^ was realized at 20.0 eV. Water, methanol and formic acid of LC-MS quality were purchased from Carlo Erba Reagents (Val de Reuil, France). The standards of HA, CA and FA were purchased from Merck Chimie SAS (Fontenay-sous-Bois, France).

**Cell culture of primary rat cortical neurons:** Primary rat cortical neurons were cultured as described by Callizot et al. (2013). Briefly, pregnant female Wistar rats (Janvier labs, Le Genest-Saint-Isle, France) at 15 days of gestation were killed using a deep anesthesia with CO_2_ chamber followed by cervical dislocation. Fetuses were collected and immediately placed in ice-cold L15 Leibovitz medium (Pan Biotech, Aidenbach, Germany) added with a 2% penicillin (10,000 U/ml) and streptomycin (10 mg/ml) solution (PS) (Pan Biotech) and 1% bovine serum albumin (BSA) (Pan Biotech). Cortex were treated for 20 min at 37°C with a trypsin-EDTA solution (Pan Biotech) at a final concentration of 0.05% trypsin and 0.02% EDTA. The dissociation was stopped by addition of Dulbecco’s modified Eagle’s medium (DMEM) with 4.5 g/L of glucose (Pan Biotech), containing DNAse I grade II (final concentration 0.5 mg/ml) (Pan Biotech) and 10% fetal calf serum (FCS) (Invitrogen *via* Thermo Fisher, Courtaboeuf, France). Cells were mechanically dissociated by three forced passages through the tip of a 10-ml pipette. Cells were then centrifuged at 515 x *g* for 10 min at 4°C. The supernatant was discarded, and the pellet was resuspended in a defined culture medium consisting of Neurobasal medium (Invitrogen,) with a 2% solution of B27 supplement (Invitrogen), 2 mM of l-glutamine (Pan Biotech), 2% of PS solution, and 10 ng/ml of brain-derived neurotrophic factor (BDNF) (Pan Biotech). Viable cells were counted in a Neubauer cytometer, using the trypan blue exclusion test. The cells were seeded at a density of 25,000 *per* well in 96-well plates (for immunostaining) and 170,000 per well in 24-well plates (for Western blot [WB] and qPCR) precoated with poly-l-lysine (Greiner, Les Ulis, France) and were cultured at 37°C in an air (95%)-CO_2_ (5%) incubator. The medium was changed every other day.

Glutamate injury: After 13 days, different doses of NSP01 extract (2.5 ng/ml, 5 ng/ml, 50 ng/ml, 500 ng/ml, 2.5 μg/ml, 5 μg/ml, 25 μg/ml, 33.3 μg/ml) or HA, CA and FA (HA 10 or 1 pM, CA 5 nM or 500 pM or 50 pM and FA 10 nM) were added to the cell culture 1 h before glutamate (Sigma-Aldrich, Saint Quentin-Fallavier France) application. The cortical neurons were injured with a 40 µM glutamate solution. After 20 min, the cells were washed-out and fresh medium, with extract or HA/CA/FA compounds, was added for 48 h additional time.

**Amyloid beta peptide injury and drug treatment:** The amyloid beta peptide _1–42_ (Aβ_1-42_) preparation was done following the procedure described by Callizot et al. (2013). Briefly, Aβ_1-42_ peptide (Bachem, Weil-am-Rhein, Germany) was dissolved in the defined culture medium mentioned above, devoid of serum, at an initial concentration of 40 μmol/L. This solution was gently agitated for 3 days at 37°C in the dark and immediately used after being properly diluted in culture medium to the concentrations of 20 μM, corresponding to 2 µM of Aβ oligomers (AβO). After 11 days, different doses of NSP01 extract (2.5 ng/ml, 5 ng/ml, 50 ng/ml, 500 ng/ml, 2.5 μg/ml, 5 μg/ml, 25 μg/ml, 33.3 μg/ml) or HA, CA and FA (HA 10 or 1 pM, CA 5 nM or 500 pM or 50 pM and FA 10 nM) were added to the cell culture 1 h before Aβ_1-42_ application. Aβ_1-42_ preparation was added to a final concentration of 20 μM diluted in control medium for 24 h.

Farnesylthiosalicylic acid (FTS; 10010501, Cayman chemical, Ann Arbor, Michigan, United States) was given 1 hour before NSP01 or HA/CA/FA compounds at final concentration of 4 µM.

**Immunostaining:** After intoxication, cells were washed with phosphate-buffered saline (PBS) (Pan Biotech) and fixed with a solution of 4% paraformaldehyde (PFA; Sigma Aldrich) in PBS, pH 7.3, for 20 min at room temperature (RT). They were washed twice again in PBS, and were then permeabilized and the non-specific sites blocked, using a solution of PBS containing 0.1% of saponin (Sigma Aldrich) and 1% of FCS, for 15 min at RT. Cells were incubated with mouse monoclonal antibody directed against microtubule-associated-protein 2 (MAP-2, Sigma Aldrich) antibody at dilution of 1/400 in PBS containing 1% of FCS, 0.1 mg/ml of saponin, for 2 h, at RT. The cells were then washed with PBS containing 1% of FCS, 0.1% of saponin, and incubated with Alexa Fluor 488 goat anti-mouse IgG secondary antibody diluted to 1/400 (Invitrogen).

**Protein analysis**: One 24 well-plate was prepared for analysis of phospho-ERK and GAPDH by WB. Sixty µL of CelLytic MT reagent (Sigma, Aldrich), supplemented with 1% of a protease and phosphatase inhibitor cocktail (PPC1010, Sigma, Aldrich), were used to lysate cells in each well. Protein content was determined by using the micro kit BCA (Pierce, Thermo Fisher Scientific). Briefly, lysates were diluted at 1/50 in PBS and mixed, in equal volume, with a micro–BCA working reagent. These solutions were incubated at 60°C for 1 h and the quantity of protein were measured at 562 nm with a spectrophotometer Nanovue (GE Healthcare, Chicago, Illinois, United States) and compared with the standard of Bovine Serum Albumin curve (BSA, Pierce, Thermo Fisher Scientific).

**WES**™ **automated WB and analysis**: All reagents (ref: SM-W002, except primary antibodies) and secondary antibodies (ref DM-001 or DM-002) were obtained from ProteinSimple®. Runs were performed according to manufacturer’s recommendations (WES™; ProteinSimple, San Jose, CA, https://www.proteinsimple.com/wes.html).

Capillaries, samples, antibodies, and matrices were loaded inside the instrument. The simple Western was run with capillaries filled with separation matrix, stacking matrix and protein samples. All conditions were incubated during 2 h with the pERK1/2 (9101S, rabbit, Ozyme, Saint-Cyr-l’Ecole, France) and GAPDH (10515025, mouse, Fisher Scientific). Each protein was evaluated independently. Capillaries were washed and incubated with HRP conjugated secondary antibodies for 1 h. After removal of unbound secondary antibody, the capillaries were incubated with the luminol-S/peroxide substrate and chemiluminescent signal was collected using the Charge-Coupled Device (CCD) camera of WES™. Data analysis was performed using the Compass Software (ProteinSimple) on WES™.

AChE activity: The activity of AChE was determined using the Acetylcholinesterase Assay Kit (Abcam, Cambridge, United Kingdom). Briefly, 50 µL of NSP01 extract (0.042, 0.42, 4.2, 42, 420 ng/ml, 4.2 and 42 μg/ml) or HA, CA, FA (1, 10, 100 and 1,000 pM, 10, 100, 100 nM) were mixed with 50 µL of reaction mixture and incubated at room temperature for 10 up to 30 min. Optical density was measured at 410 nm wavelength by GloMax® spectrophotometer (Promega, Charbonnières-les-Bains, France). Three samples (biological replicates) *per* condition were analyzed.

Statistical analyses: All data were expressed in percentage of control conditions (no injury, no glutamate/no Aβ = 100%). All values are expressed as mean ± SEM. Graphs and statistical analyses were performed using one-way ANOVA followed by LSD Fisher’s test or Dunnett’s test when allowed using GraphPad Prism, version 7.04 (GraphPad software Inc., La Jolla, CA, United States). *p* < 0.05 was considered as significant. The synergistic score was calculated according to the Loewe’s equation.

## Results

### NSP01, a Standardized Green Extract of *H. serrata*

Inspired by the tradition, we applied a one-step extraction method to generate a standardized and reproductible extract of *H. serrata* (NSP01, Supplementary Figure S1). In order to maintain a green extraction method, we used only water as solvent and the assistance of microwave technology to speed up the extraction process. In parallel, a traditional extract of *H. serrata* (NSP01-TE) was prepared using decoction method. The extraction yields were: 15 and 20% (m/m) for NSP01-TE and NSP01, respectively.

**FIGURE 1:**
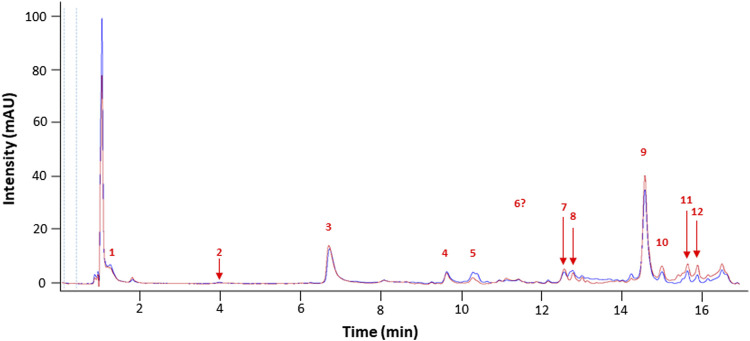
Representative chromatograms of NSP01-TE (blue) and NSP01 (red) *H. serrata* extracts using UHPLC-DAD at 310 nm. Peak numbers refer to those implemented in Table 1.

Chromatograms (UHPLC-DAD 310 nm, (Wu and Gu, 2006)) showed that both extracts displayed a similar profile, suggesting a very close phytochemical composition (Figure 1).

Identification of alkaloid compounds (positive mode) and phenolic compounds (negative mode) was performed using an UHPLC-DAD-ESI-MS/MS method and the results are listed in Table 1.

**TABLE 1:** Phytochemical composition of NSP01-TE and NSP01 using UHPLC-DAD-ESI-MS/MS analysis.

Peak	_tR_ (min)	Adduct	Calculated *m/z*	Experimental *m/z*	Mass accuracy ∆m (mDa)	Experimental MS^2^ m/z	Identification	Fragment identification	Fragment structure
1	1.3	[M−H]^−^	315.0711	315.0718	0.7	153.0185	Protocatechuic acid-O-hexoside	Protocatechuic acid	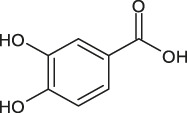
2	4.0	[M−H]^−^	153.0182	153.0192	1.0	109.0294	Protocatechuic acid	1,2-benzendiol	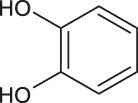
3	6.7	[M+H]^+^	243.1492	243.1487	0.5	226.1233	Hup A	Desamino-huperzine A	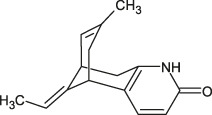
4	9.7	[M−H]^−^	179.0339	179.0347	0.8	135.0449	Caffeic acid	3–4,dihydroxystyrene	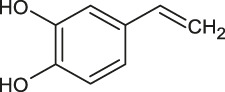
5	10.3	[M−H]^−^	-	395.0964	NA	193.0498	Unknown glucuronide	Unknown	Unknown
						175.0395		Unknown	Unknown
						201.0397		Unknown	Unknown
						219.0501		Unknown	Unknown
6	12.0	[M−H]^−^	353.0867	353.0875	0.8	191.0557	Chlorogenic acid^a^	1,3,4,5-tetrahydroxycyclohexanecarboxylic acid	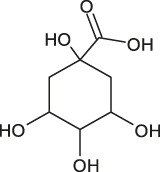
						161.0249		*unamed*	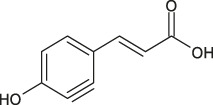
						179.0357		Caffeic acid	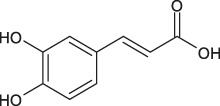
7	12.6	[M−H]^−^	-	308.0769	NA	149.0603	Unknown	Unknown	Unknown
						192.0657		Unknown	Unknown
						134.0368		Unknown	Unknown
8	12.8	[M−H]^−^	-	473.1286	NA	329.0866	Unknown	Unknown	Unknown
						167.0341		Unknown	Unknown
9	14.7	[M−H]^−^	193.0495	193.0502	0.7	134.0367	Ferulic acid	3–4,dihydroxystyrene (radical)	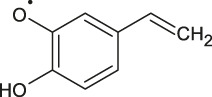
						178.0264		Caffeic acid (radical)	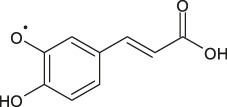
10	15.0	[M−H]^−^	-	399.0932	NA	193.0497	Unknown phenolic acid	Unknown	Unknown
						167.0341		Unknown	Unknown
						237.0394		Unknown	Unknown
11	15.7	[M−H]^−^	-	395.0971	NA	193.0499	Unknown glucuronide	Unknown	Unknown
						219.0504		Unknown	Unknown
						201.0397		Unknown	Unknown
						175.0395		Unknown	Unknown
12	15.9	[M−H]^−^	-	501.1606	NA	195.0655	Unknown	Unknown	Unknown
						357.1182		Unknown	Unknown
						399.1284		Unknown	Unknown

a The intensity of compound 6 in both extracts is very low. However, the fragmentation was possible in NSP01-TE and NSP01 extract and the fragment ions of compound 6 are presented from NSP01-TE extract fragmentation.

A fragment ion of Compound 1, with *m/z* 153.0185, could correspond to Compound 2, which was identified as protocatechuic acid. The 162 Da difference with the parent ion suggested the loss of a glucose residue (Spínola et al., 2015) and confirmed the identity of Compound 1, as protocatechuic acid glucoside (R*t* 1.3 min and parent ion *m/z* 315.0711 [M-H]^-^). Compound 2 was identified as Protocatechuic acid since it eluted at R*t* 4.0 min with *m/z* 153.0182 for parent ion [M-H]^-^ and *m/z* 109.0294 for fragment ion. The identification hypothesis confirmed by comparing the experimental spectra with the spectra of Protocatechuic acid in the database of PubChem. Compound 3 was one of the major components of NSP01-TE and NSP01 extracts. This compound with *m/z* 243.1487 for parent ion [M + H]^+^and *m/z* 226.1233 for fragment ion (corresponding to a loss of NH_3_) was identified as HA (Yuan Sq, 2000). Compound 4 with *m/z* 179.0339 for the parent ion [M-H]^-^ was also a major compound in NSP01-TE and NSP01 extracts. The presence of a fragment ion with *m/z* 135.0449, corresponding to a loss of 44 Da, characteristic of CO_2_ loss from the carboxylic acid group of phenolic acids (Ben Said et al., 2017), was observed. The compound was identified as CA (Sapozhnikova et al., 2014). Compound 5 and 11 with the parent ions at *m/z* 395.0964 and 395.0971, respectively, were supposed to be two isomers. Indeed, the same fragment ions were obtained for both compounds: at *m/z* 193.0498/193.0499, 175.0395/175.0395, 201.0397/201.0397 and 219.0501/219.0504 for compound 5**/**compound 11, respectively. The loss of 176 Da could correspond to a loss of glucuronic acid. Because the negative charge was retained on the glucuronide moiety, an abundant glucuronate fragment (*m/z* 193) was often seen in the negative-ion mode with a subsequent dehydration, yielding to a less abundant ion at *m/z* 175 (Docampo et al., 2017). Although, these two compounds were not formally identified, they were assumed to be glucuronic acid derivatives. Compound 6 was a minor compound with parent ion at *m/z* 353.0875 [M-H]^-^. A loss of 162 Da was observed and could correspond to a loss of either glucose moiety or caffeoyl moiety. The spectrum, comprising three major fragment ions at *m/z* 161.0249, 179.0357, and 191.0557, was compared to spectrum of chlorogenic acid spectrum from the database of PubChem.- The comparison confirmed the identification as chlorogenic acid (Sapozhnikova et al., 2014). No structure could be proposed for the compounds 7, 8 and 12 with parent ions at *m/z* 308.0769, 473.1286 and 501.1606, respectively. Compound 9, a major compound of both extracts, with parent ion at *m/z* 193.0495 [M-H]^-^ was identified as FA by comparing experimental mass spectrum to those of FA from the database of PubChem (Sapozhnikova et al., 2014). A fragment of compound 10, with parent ion at *m/z* 399.0932, showed a loss of 162 Da, that could correspond either to a loss of glucose moiety, or to a loss of caffeoyl moiety. The difference of mass between two other fragments (*m/z* 237.0394 and *m/z* 193.0497) was 44 Da, a characteristic CO_2_ loss from the carboxylic acid group of phenolic acids (Ben Said et al., 2017). Therefore, the compound 10 was identified as a phenolic acid either glycosylated or caffeoylated.

Three major compounds were identified namely HA, CA and FA. Mean contents were 0.17, 0.03 and 0.08% (HA, CA and FA, respectively) as determined on three NSP01 batches. Since these three components were the major ones, we decided to study firstly these molecules.

### Neuroprotective Activity of the NSP01 Extracts

Glutamatergic dysfunction linked to Ca^2+^ dyshomeostasis plays a great importance in pathophysiology of AD (Benarroch, 2018). Changes in *N*-methyl-d-aspartate receptors (NMDARs) appear to be involved in synaptic dysfunction in early stages of AD. In this regard, selective inhibition of NMDARs-mediated excitotoxicity alone may help to slow down the progression of synaptic disruption in AD (Conway, 2020).

**FIGURE 2:**
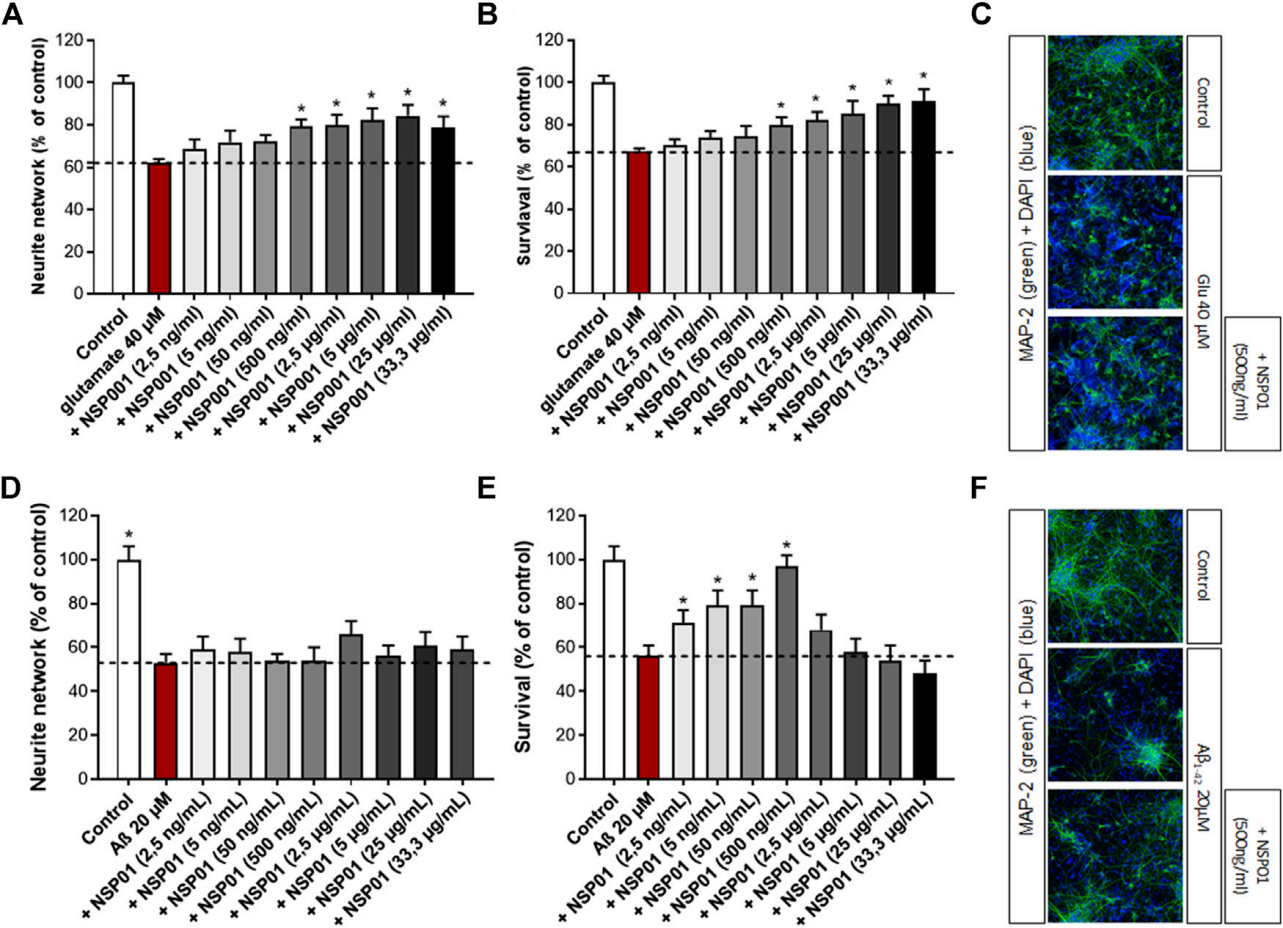
Neuroprotective activity of NSP01 *H. serrata* extracts. Neurite network and cell survival were measured in primary rat cortical neurons by immunohistochemistry (MAP-2 antibody). **(A,B)**, immunohistochemical quantification of MAP-2 in neurons injured with glutamate (40 μM, 20 min) and pre-treated with several doses of NSP01 extract for 48 h. **(C)**, representative MAP-2 staining (green) of primary rat cortical neurons injured with 40 µM of glutamate. 500 ng/ml of NSP01, containing the equivalent of 2,9 nM HA, 0,7 nM of CA and 1,7 nM of FA, represents the first effective dose leading to neuroprotection. **(D–F)**, immunohistochemical quantification and pictures of MAP-2 in neurons injured with Aβ_1-42_ oligomers (20 μM, 24 h) and pre-incubated with several doses of NSP01 extract (1 h). The best effect was obtained after treatment with 500 ng/ml of extract. All values are expressed as mean ± SEM (standard error of the mean). *p < 0.05 vs. control.

We firstly aimed to assess the effects of NSP01 extract (generated by our laboratory with the one-step extraction method) on primary neuronal cultures injured with glutamate. In our model, cortical neurons were exposed to high dose of glutamate (40 μM, 20 min), which resulted in enhanced localized vulnerability of neurons in a manner consistent with AD neuropathology. Thus, a significant decrease of neuron survival (by ∼ 32%) and their neurite network (by ∼38%) was observed (Figures 2A,B, *red bar*). Importantly, the previous treatment with NSP01 (1 h) was able to significantly protect neurons and neurite network from glutamate injury (Figures 2A,B, *grey bars* and C).

We also tested the effect of NSP01 extract in another AD model, where cortical neurons were cultured in presence of Aβ oligomers (Callizot et al., 2013). As expected, the application of Aβ_1-42_ induced neurites reduction and neuronal death (Figures 2D,E, *red bar*). Also, in this model, NSP01 was able to protect neurons survival, with the higher effect reached at 500 ng (Figures 2E,F) but not neurite network (Figure 2D) whichever the dose tested.

### Synergy of the Three NSP01 Main Components (HA, CA, FA) and Neuroprotective Effects

**FIGURE 3:**
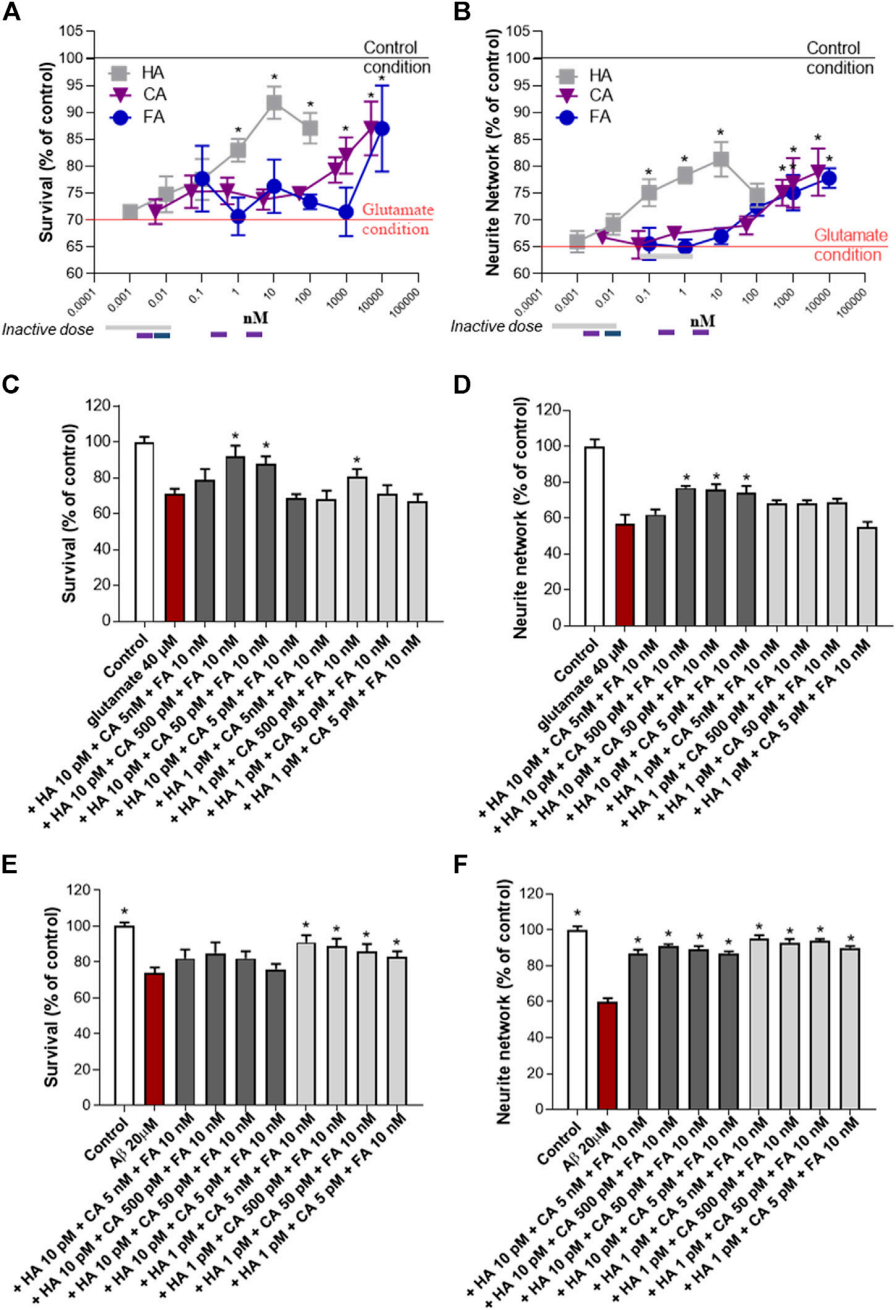
HA, CA and FA synergistically promote neuroprotection after glutamatergic or amyloid stress. Neuroprotective activity of HA, CA and FA alone **(A, B)** or a combination of them on neuron survival and neurite network was measured by MAP-2 immunohistochemistry after glutamate **(C, D)** or AβO **(E, F)** injury. All values are expressed as mean ± SEM (standard error of the mean). *p < 0.05 vs. control; ^#^ *p* < 0.05 vs. glutamate.

The NSP01 chemical profile showed the presence of three main component: the HA, the CA and the FA. Therefore, we tested the neuroprotective effects of these molecules on primary culture of cortical neurons exposed to glutamate. In our model, glutamate (40 μM, 20 min) induced a significant neuronal death and a large loss of neurite (Figures 3A,B, *red baseline*)*.* Significant protective effect on survival and neurite network was observed for the three molecules when applied alone (Figures 3A,B). Thus, more than 1,000 nM of CA or FA (blue and violet lines) are needed to maintain neuron morphology to that of control condition (*black baseline*) and HA positive effects were observed from the dose of 0.1 nM and above (grey line). Interestingly, the association of these three molecules at non-active concentrations showed a significant protective effect (Figures 3C,D, *grey bars*). Thus, HA/CA/FA at 10 pM/500 pM/10 nM and 10 pM/50 pM/10 nM improved neuron survival (>80% of survival) (Figure 3C, *dark grey bars*) and neurite network (Figure 3D, *dark grey bars*). The 10 pM/5 pM/10 nM concentration was only found effective on neurite network. Importantly, the same doses of CA + FA were ineffective on neurons survival and neurite network when combined in absence of HA (Supplementary Figure S2 **)**. The synergistic mixture was also tested in cortical neurons exposed to Aβ_1-42_ preparation. In this model, all tested concentrations of HA/CA/FA mixture were able to protect from Aβ injuries (Figures 3E,F).

In conclusion, low concentrations of HA, CA and FA which normally were ineffective after glutamate or Aβ intoxication, were able to improve neuron survival and neurite network when administered together proving the synergistic action of the three molecules. The synergistic score was equal to 0.0015 (Supplementary Figure S3 **)**.

### CA and FA don’t Potentiate HA-dependent AChE Inhibition

HA is a well-known competitive and reversible AChE inhibitor (Wang and Xi, 2005). It is structurally similar to acetylcholine and acts on AChE in a different way than other inhibitors, binding to the peripheric anionic site (Wong et al., 2003; Xu et al., 2003). HA side effects include vomiting, diarrhea, dizziness, headache, insomnia, restlessness, excitement, and fatigue (Li et al., 2008). Therefore, we evaluated the effect of FA and CA on AChE enzymatic activity to exclude any effect that could work in synergy with HA and increase the AChE inhibition.

**FIGURE 4:**
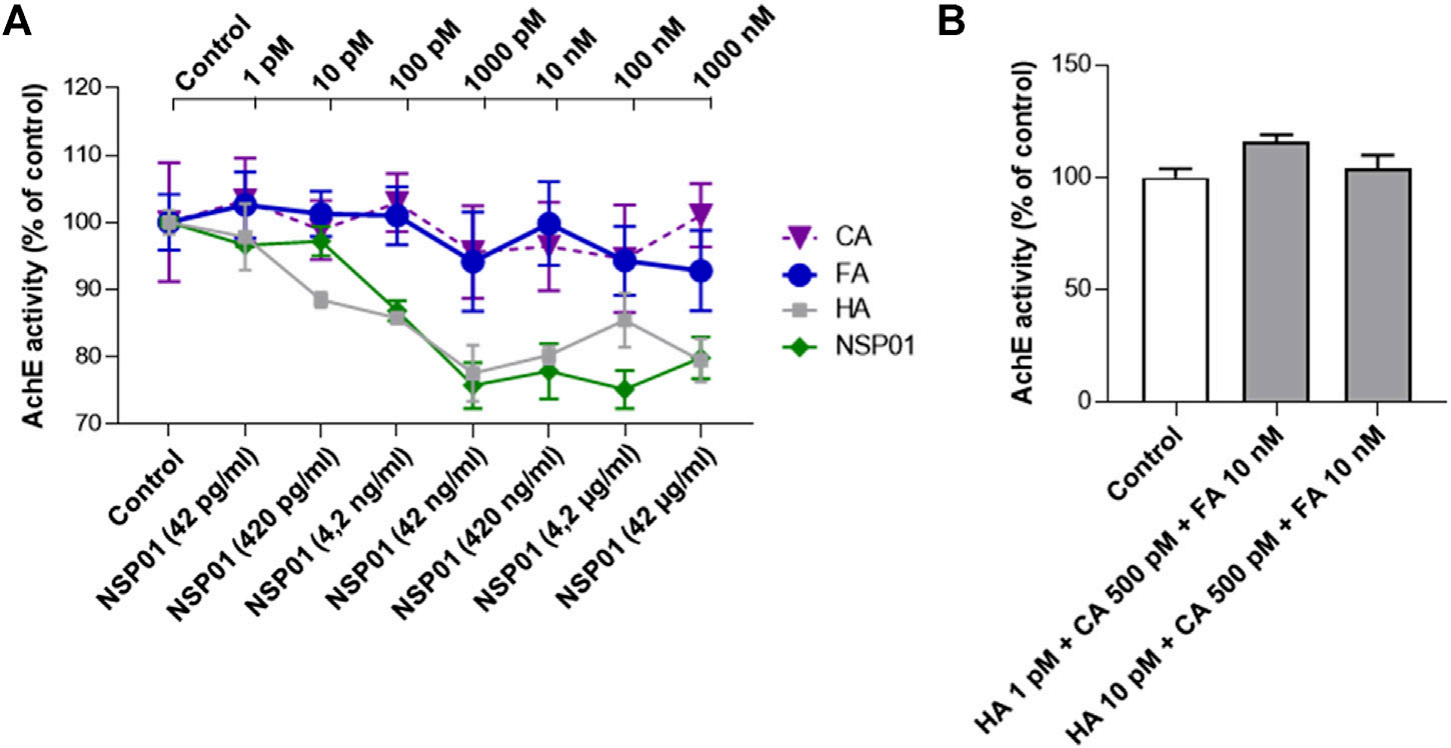
**(A)**, AChE activity in increasing doses of NSP01 extract and HA, CA and FA. In the abscissae the concentrations of extract tested to determine the inhibitory effect on AChE; in the upper part of the graph the concentrations of HA, CA and FA. Six samples (biological replicates) per condition were analyzed. **(B)**, effect of HA/AC/FA mixtures on AChE enzymatic activity. Measurements were done on three biological replicates.

As expected, increasing doses of HA (grey line) or NSP01(green line) progressively decreased AChE activity (Figure 4A). Importantly, all doses of CA (violet line) or FA (blue line), found to be neuroprotective when associated with HA, do not potentiate HA-dependent AChE inhibition.

Finally, we measured AChE activity in presence of HA/AC/FA mixtures at concentrations previously proven to be inactive alone but neuroprotective when administrated together (Figure 4B). The samples tested showed no difference in AChE activity in respect with the control condition.

In conclusion, our results excluded any potentiation of AChE activity by NSP01 extract.

### Mechanism of Action of NSP01 and HA/CA/FA Mixture

**FIGURE 5:**
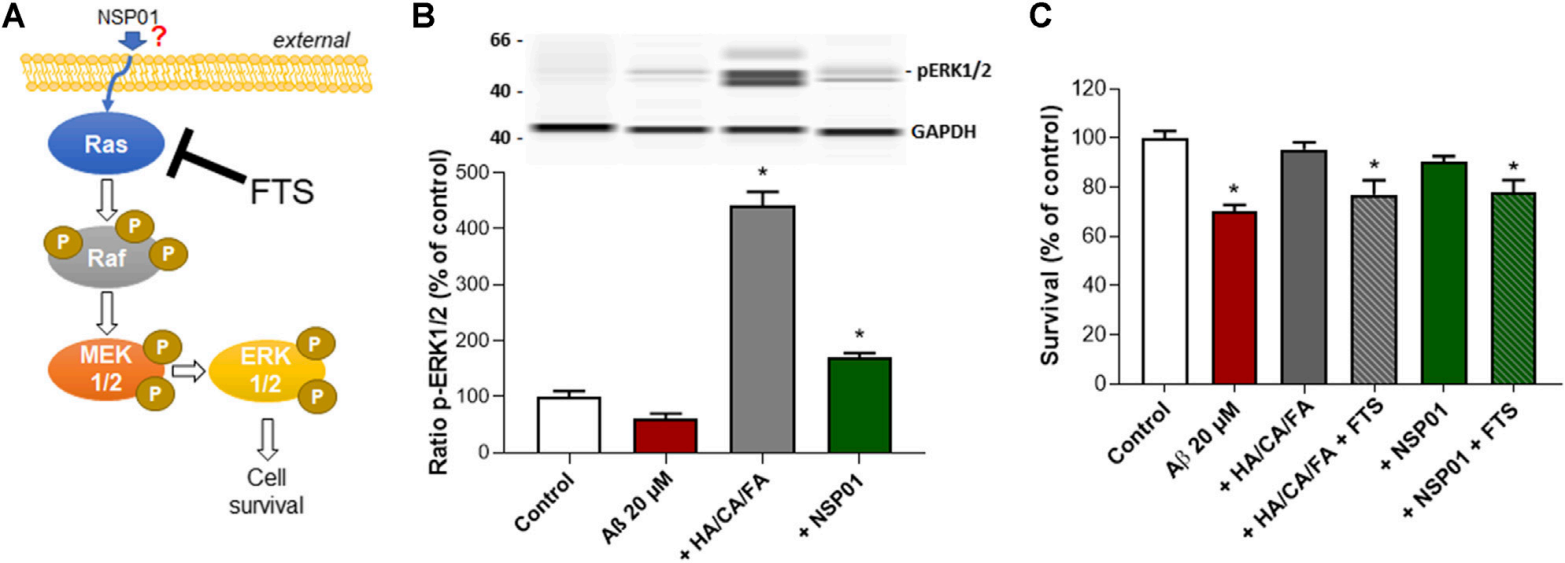
Inhibition of Ras pathway prevent NSP01 or HA/CA/FA neuroprotection. **(A)**, schematic representation of Ras/Raf/MEK/ERK1/2 pathway and FTS site of action. Briefly, growth factors or mitogens lead to the activation of specific receptors on the plasma membrane which in turn transmit the activating signals to Ras. Ras interacts and phosphorylates a wide range of downstream effector proteins, including Raf proteins (isoforms of the serine/threonine kinase). MEK1/2 is then phosphorylated at two serine residues and subsequently phosphorylates ERK1/2 on both threonine and tyrosine. Activated ERK1/2 proteins translocate to the nucleus where they activate multiple transcription factors causing changes in cell proliferation and survival. **(B)**, WB of ERK1/2 phosphorylation normalized to GAPDH. **(C)**, quantification of MAP-2 staining in cortical neurons exposed to 20 µM of Aβ and treated with NSP01 (25 μg/ml) or HA 1 pM + CA 500 pM + FA 10 nM mixture with or without FTS (4 µM). All values are expressed as mean ± SEM (standard error of the mean). *p < 0.05 vs. control.

The mitogen-activated protein kinase ERK has been shown to promote cell survival by a dual mechanism comprising the modification and inactivation of cell death machinery and the increased transcription of pro-survival genes (Kolch, 2005). The Ras–Raf–MEK–ERK signaling cascade is the most known pathway controlling cell proliferation after extracellular growth factors, cytokines or mitogens stimulation (Mebratu and Tesfaigzi, 2009; Figure 5A). Interestingly, both NSP01 and HA/CA/FA were able to increase ERK phosphorylation in cortical neurons after Aβ_1-42_ intoxication (Figure 5B). Thus, we hypothesized that ERK pathway could be responsible for NSP01 neuroprotective effects.

To this end, we treated cortical neurons, previously injured with Aβ_1-42_ and exposed to NSP01 or HA/CA/FA, with FTS. FTS acts as functional Ras antagonist thus blocking Raf–MEK–ERK cascade (Schmukler et al., 2017). Inhibition of Ras/Raf/MEK/ERK pathway completely blocked the positive effect of NSP01 and HA/CA/FA on neurons survival (Figure 5C), suggesting a possible mechanism of action passing through the ERK signaling pathway. However, how NSP01 targets ERK pathway is still unknown.

## Discussion

For centuries, the herbal preparation of *H. serrata,* also known as *L. serrata*, was used in popular medicine to cure/treat many disorders such as contusions, swelling, schizophrenia and cognition dysfunction in the elderly. In China and in most of Asian populations, the herbal decoction of this plant is still safely used (Wang et al., 2010; Andrade et al., 2019). *H. serrata* contains triterpenoids and various alkaloids (0.2%), including lycodoline, lycoclavine, serratinine, and huperzines. HA is the most studied alkaloid found in *H*. *serrata*, due to its ability to reverse or attenuate the cognitive deficits found in some animal models. Indeed, HA reversibly inhibits AChE *in vitro* and *in vivo* with a potency much higher than huperzine B, donepezil or rivastigmine (Wang et al., 2006b). In addition, it has been observed that HA is endowed with neuroprotective properties against Aβ injury, oxygen-glucose deprivation, glutamate and free radical-induced cytotoxicity (Zhang and Tang, 2006; Gao et al., 2009). Also, HA is an NMDA receptor antagonist (Gordon et al., 2001), it reduces the apoptosis by inhibiting the mitochondria-caspase pathway and has neurotrophic properties (Wang and Xi, 2005).

Nevertheless, the HA-related memory improvement in AD patients seen in the multiple clinical trials (performed mostly in China), was contrasted by the appearance of few adverse effects (Li et al., 2008; Yang et al., 2013). Therefore, it is difficult to draw definite conclusions regarding its real interest for treating AD. Even if HA is one of the safest of the AChE inhibitors, high dosage (from 0.2 to 0.8 mg daily) is needed to reach a significant improvement of cognition. This often results in cholinergic reaction and adverse effects such as sweating, nausea, vomiting, dizziness, hypertension, headache, tachycardia, and insomnia (Little et al., 2008).

Facing the discrepancies between the lack of HA safety at efficient doses and the safe use of the traditional herbal medicine, we decided to conduct an inquest to find an explanation, hypothesizing that the efficacy of the traditional extract was not solely based on HA, but also on other components. In order to standardize the herbal decoction, we used a green extractive process, inspired by the traditional one. Our protocol allowed the extraction of a mixture, that we called NSP01, chemically comparable to the extract obtained through traditional decoction. The chemical profile of NSP01 raised not only HA, as component, but also two major phenols acid: CA and FA.

FA is an important component of many medicinal herbs. It has anti-inflammatory, antioxidant, and neuroprotective properties (Sultana et al., 2005; Picone et al., 2009; Mori et al., 2013). Also, CA has neuroprotective actions which encompasses a wide spectrum of mechanisms, going from protection of dopaminergic neurons against inflammation injury (Kurauchi et al., 2012) to induction of neurite outgrowth (Yang et al., 2015) and neurogenesis (Fu et al., 2013; Moosavi et al., 2017). Nevertheless, the cellular mechanisms by which CA induces all the broad range of neurotrophic effects are not fully understood.

In this study, we showed that low doses of CA and FA, demonstrated to be inactive in our systems, became neuroprotective when they were associated with low concentrations of HA. Thus, a pre-incubation with 1 pM of HA, 500 pM of AC and 10 nM of FA was able to protect neurons injured with glutamate or Aβ_1-42,_ in two *in vitro* models of AD. More interestingly, when the molecules were associated in pairs, at these similar doses no neuroprotection was observed. These results were in favor of a synergistic action between the three molecules rather than any additional effect. Moreover, the calculation of the synergy score of Loewe (Merlin, 1994; Roell et al., 2017) showed un index below one proving the synergy among the molecules.

In light with these results, we believed that the neuroprotective effect of NSP01 is probably linked to HA/CA/FA combination.

Although the synergistic effect at low doses of NSP01 is not strong and evident in the glutamatergic model, it is clear in cells stressed by Aβ oligomers. Thus, 2.5 ng/ml of NSP01 (which contains only 17.5 pM HA, 4.2 pM CA and 10.3 pM FA) has a significant benefit on cell survival after Aβ intoxication. These data provide evidence for a different mode of action of NSP01 in the two models and merit further exploration to elucidate how the NSP01 extract specifically acts.

We also explored the pharmacological pathways involved in NSP01 or HA/CA/FA activities and showed that their neuroprotective effects depended from the Ras–Raf–MEK–ERK pathway activation. Thus, the inhibition of Ras by a specific antagonist (FTS), completely reversed their beneficial effect on neuron survival after Aβ intoxication.

The Ras–Raf–MEK–ERK pathway is an evolutionary conserved pathway involved in the control of fundamental processes that include cell proliferation, differentiation, survival, apoptosis and nervous system plasticity (Curtis and Finkbeiner, 1999; O’Neill and Kolch, 2004; Keshet et al., 2010; Plotnikov et al., 2011; Napoli et al., 2012). The dysregulation of this cascade is generally associated with neurodegenerative disorders, including AD (Subramaniam and Unsicker, 2010; Rai et al., 2019).

The Ras/Raf/MEK/ERK cascade reaction is activated by both survival and death signals (Subramaniam and Unsicker, 2010); however the mechanisms which regulate such differential scenarios are not yet clear. Among the various stimuli, the activation of the surface receptor TrkB (tyrosine kinase B), by its high affinity ligand BDNF (brain-derived neurotrophic factor), induces the activation of the Ras/Raf/MEK/ERK cascade, which in turn increase the expression of pro-survival genes (Subramaniam and Unsicker, 2010; Bathina and Das, 2015). Thus, the transient activation of ERK1/2 observed upon BDNF stimulation protects hippocampal neurons from glutamate toxicity (Almeida et al., 2005). Also, the nerve growth factor (NGF) mediates sustained ERK1/2 activity leading to neurite outgrowth and cell survival in PC12 cells (Marshall, 1995). In the same way, the transforming growth factors-β (TGF-β) exerts its major biological role, the transition from epithelial to mesenchymal phenotype necessary for embryonic development (Zhang, 2009).

Importantly, HA is able to regulate and induce NGF production in cultured astrocytes (Tang et al., 2005a) and to protect SH-SY5Y cells against oxidative stress damage by promoting NGF production (Tang et al., 2005b). Also *in vivo*, HA has been proved to persistently increase NGF, BDNF and TGF-β1 mRNA levels in cerebral cortex and hippocampus of mice injured with ischemia and reperfusion (Wang et al., 2006a). Interestingly, the HA-dependent regulation on neurotrophic factors was associated to ERK 1/2 phosphorylation and behavioral damage amelioration (Wang et al., 2006b). The implication of BDNF or other neurotrophins in the mechanism of action of NSP01 is still under investigation. However, preliminary data from injured neurons did not show any robust increase of BDNF mRNA or protein levels after NSP01 48 h treatment (data not shown). Nevertheless, it is important to underline that the doses of HA explored in our studies were significantly lower than the doses used in all previous works. Also CA and FA attenuate BDNF downregulation and exert antidepressant-like effects in mouse (Takeda et al., 2006; Dzitoyeva et al., 2008; Liu et al., 2017), however our results suggest the existence of BDNF-independent pathways that may synergize and mediate the NSP01 trophic effects.

The ERK1/2 pathway is the classical and the best studied one; however, it is part of a larger family of MAP kinases (MAPKs) which comprise p38; the c-Jun amino terminal kinase (JNK); and ERK5. ERK1/2 pathway typically transduces growth factor signals that lead to cell differentiation or proliferation, whereas cytokines and stress signals activate the JNKs and p38 MAPK pathways, which exert antagonistic effects on cell proliferation and survival (Wagner and Nebreda, 2009).

CA strongly inhibits phosphorylation of JNK and p38 MAPK by inhibiting Fyn kinase activity (Onodera et al., 2015). As consequence, the prostaglandin synthase 2 (COX-2) is downregulated and inflammation reduced (Spangler, 1996). Moreover, both CA and FA increases cell viability and attenuates H_2_O_2_-DNA damages by activating ERK signal pathway (Reunanen et al., 2002; Pluemsamran et al., 2012; Li et al., 2015).

Therefore, the neurotrophic effects of NSP01 may occur through its anti-inflammatory, immunomodulatory, and antioxidant properties (CA and FA) and its induced pro-survival properties on neurons (BDNF).

Due to its complex mechanism of action, further exploration of JNK and ERK1/2 activity is required. Inhibition of Ras by FTS decreased ERK phosphorylation, but it may also affect JNK activation. Obviously, MEK inhibitors need to be tested to confirm the involvement of the ERK pathway (Peng et al., 2007).

Finally, the three main components of NSP01 did not increase AChE inhibition. Thus, the beneficial effects of NSP01, mimicking the traditional *H. serrata* decoction, were observed at lower doses than the usual efficient concentrations of HA. This allows to reduce the potential cholinergic adverse effect of this molecule.

In conclusion, our findings shed light on the critical role of traditional medicine and underline the importance in evaluating the crude extracts vs the active constituents of a plant to improve pharmacology knowledge and diseases treatments.

## Data Availability

The original contributions presented in the study are included in the article/Supplementary Material, further inquiries can be directed to the corresponding author.
