# Supplementary material for: Huperzia serrata Extract ‘NSP01’ With Neuroprotective Effects-Potential Synergies of Huperzine A and Polyphenols
Source: Front Pharmacol. 2021 Aug 30;12:681532. doi: 10.3389/fphar.2021.681532 (PMC8435632; doi:10.3389/fphar.2021.681532)
Supplement: Supplementary file 1 [file DataSheet1.docx]

**NSP01, a green extract of *Huperzia serrata* with neuroprotective effects related to synergistic action of Huperzine A and polyphenols.**

**Supplementary figure 1**:


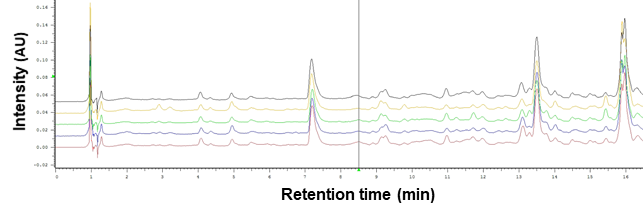


**Supplementary figure 2**:


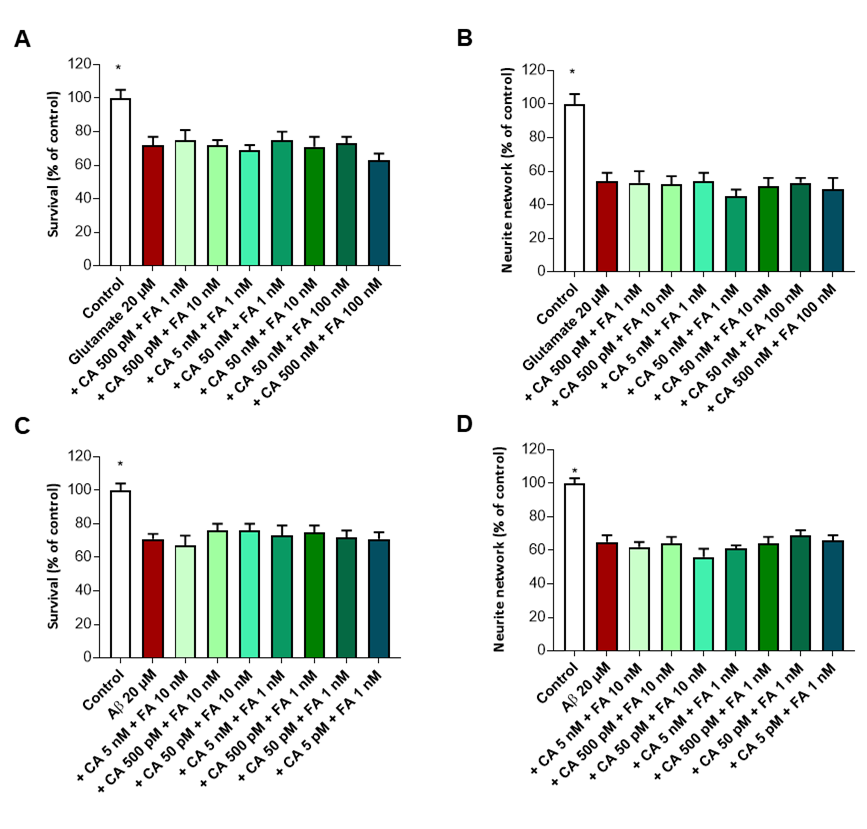


**The combination of inactive doses of CA and FA do not promote neuroprotection after glutamatergic or amyloid stress**. Neurite network and cell survival were measured in primary rat cortical neurons by immunohistochemistry (MAP-2 antibody). **A** and **B**, immunohistochemical quantification of MAP-2 in neurons injured with glutamate (40 µM, 20 min) and pre-treated with several doses of CA + FA for 48 hours. **C** and **D**, MAP-2 staining in neurons injured with Aβ_1-42_ oligomers (20 μM, 24 h) and pre-incubated with several doses of CA + FA (1 hour). All values are expressed as mean +/- SEM (standard error of the mean). * P < 0.05 vs control.

**Supplementary figure 3**:


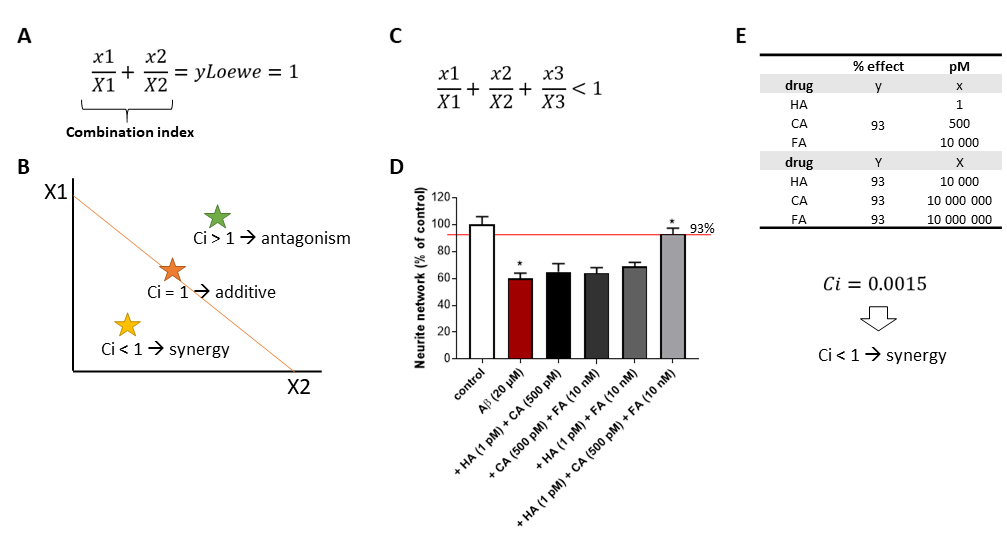


To understand the relationship among HA, CA and FA, we used the reference model based on “Loewe additivity”. In this model (**equation A**), X1 and X2 are doses of compound 1 and 2 required to produce un effect “*yLoewe”* when given alone*. x1* and *x2* are lower doses of the same drugs able to produce the *yLoewe* effect when combined. In a Cartesian system, the equation A, is represented as a straight line connecting *X1* and *X2* doses (**B**). The line, also called Isobole of additivity, represents the “additive” effect of the two drugs. When the Ci is lower than 1, it means that the effect *y* is reached by the combination of lower doses (*x1* and *x2)* of the same drugs, so we can define the new combination synergic. We have an antagonism effect when Ci is greater than 1. Finally, the synergism among HA, CA and FA must respect the equation in **C**. **D**, representative results obtained in primary rat cortical neurons injured with Aβ_1-42_ oligomers (20 μM, 24 h) and pre-incubated with specific doses of HA + AC, AC + AF, HA + AF and HA/AC/AF mixture. **E**, 1, 500 and 10 000 pM are the x1, x2 and x3 concentrations of HA, CA and FA respectively, which give rise to 93% of effect (y, neurite network preservation) when administrated together in the Aβ model. 10, 10 000 nM are the doses of HA, CA and FA able to produce the same effect (Y) when administrated separately (**Figure 3 B**). The substitution of those data in the equation in C, gives rise to an Ci of 0,0015.
